# Mitochondrial and Nuclear DNA Analyses of *Rhipicephalus microplus* from Mizoram, Northeast India: Insights into Genetic Diversity and Endosymbiont

**DOI:** 10.3390/genes16101216

**Published:** 2025-10-15

**Authors:** Khawlhring Lalawmpuii, Siju Susan Jacob, Thingujam Chaa Tolenkhomba, Parthasarathi Behera, Joy Lalmuanpuia, Hmar Tlawmte Lalremsanga, Khawlhring Lalrintluanga, Chhakchhuak Lalchhandama, Lal Biakzuala, Hmar Lalrinkima

**Affiliations:** 1 College of Veterinary Sciences & Animal Husbandry, Central Agricultural University Imphal, Selesih, Aizawl 796014, Mizoram, India; omomi44585@gmail.com (K.L.); drkhomba10@gmail.com (T.C.T.); partha_vet@yahoo.co.in (P.B.); joympvet@gmail.com (J.L.); drtluanga40@gmail.com (K.L.); vet2tox@gmail.com (C.L.); 2 ICAR-National Institute of Veterinary Epidemiology and Disease Informatics, Yelahanka, Bengaluru 560064, Karnataka, India; siju.jacob@icar.org.in; 3 Developmental Biology and Herpetology Laboratory, Department of Zoology, Mizoram University, Aizawl 796004, Mizoram, India; htlrsa@yahoo.co.in

**Keywords:** *Rhipicephalus microplus*, Northeast Hills of India, COX1, 16S rDNA, ITS-2, 18S rDNA, phylogeny

## Abstract

**Background/Objectives**: In this study, we conducted molecular identification of *R.microplus* and explored the genetic diversity of *R. microplus* for the first time in Mizoram, a Northeastern Hill (NEH) state of India bordering Myanmar. **Methods**: To assess genetic variation and evolutionary relationships, we employed phylogenetic analyses, genetic divergence metrics, and haplotype network construction based on mitochondrial (COX1 and 16S rDNA) and nuclear (ITS-2 and 18S rDNA) markers. Additionally, multivariate Principal Coordinate Analysis (PCoA) was used to visualize genetic differentiation among *R. microplus* populations. **Results**: Our analyses indicated that populations of *R. microplus sensu lato* from India, Bangladesh, and Pakistan form a closely related matrilineal lineage distinct from *R. microplus sensu stricto*, clustering within clade C of the COX1-based phylogeny. Globally, 24 COX1 haplotypes were recovered, with 1 haplotype identified in India. The Mizoram population exhibited a single 16S rDNA haplotype; however, intraspecific divergence was evident across India, with seven matrilineal haplotypes detected and nineteen globally. Further, five haplotypes were identified within *R. microplus* using the ITS-2 marker, while five haplotypes were observed within the *Rhipicephalus* genus using the 18S rDNA marker. Moreover, this study revealed the presence of *Coxiella-*like endosymbionts in 95% of the tick specimens analyzed. **Conclusions**: This study fills a critical knowledge gap by providing the first molecular documentation of tick diversity in Mizoram, a strategic region along the Indo–Myanmar border, and offers novel insights into the phylogeography and symbiotic associations of *R. microplus* and related tick taxa.

## 1. Introduction

Ticks are known to serve as vectors for highly pathogenic microorganisms that adversely affect both humans and animals. Moreover, tick infestations induce stress and anemia in cattle, significantly reducing overall productivity [1,2]. In India, the economic cost for managing tick and tick-borne diseases (TTBDs) in the dairy sector is estimated to be approximately USD 787.63 million annually. Increasing global connectivity has heightened the risk of introducing new diseases and their vectors into previously unaffected areas, posing a serious threat to regional livestock health [3].

Mizoram, located in north-eastern India (between 21°56′ N to 24°31′ N latitude and 92°16′ E to 93°26′ E longitude), shares international borders with Myanmar to the east and south and Bangladesh to the west. Importation of cattle from Myanmar into Mizoram was halted after October 2022 (*Northeast Today* 22 October 2022) [4]. With reference to this, molecular phylogenetic analysis revealed that the Porcine Reproductive and Respiratory Syndrome (PRRS) virus affecting pigs in Mizoram is genetically closer to the strains found in China and Vietnam than in other regions of India, hinting at possible trans-boundary transmission of the disease [5].

The Indo–Myanmar–Bangladesh region is recognized as one of India’s four biodiversity hotspots. Although Brahma et al. (2014) [6] have examined tick fauna in Assam, their study did not include samples from other north-eastern states of India. In Arunachal Pradesh, five tick species, *R. microplus*, *Amblyomma integrum*, *Haemaphysalis longicornis*, *H. bispinosa* and *Ixodes ovatus*, were identified from three districts based on morphological and molecular approaches, and *R. microplus* was found to be the most prevalent [7].

In contrast, research on tick diversity in Mizoram remains limited. Apart from the morphological identification of *A. testudinarium* [8] and reports of haemoprotozoan pathogens transmitted by *Rhipicephalus* spp. [9], there has been no systematic investigation into tick species composition, genetic variation, or phylogenetic relationships in the state.

The invasive tick species *R. microplus* is particularly concerning due to its short life cycle and increasing resistance to acaricides, which is attributed to its high adaptability and rapid evolution [10]. Previous studies based on the Cytochrome C Oxidase subunit I (COX1) gene have identified three major clades of *R. microplus*: clade A, closely related to *R. australis*, is found in parts of Southeast Asia, Africa, and the Americas; clade B is exclusively found in China; clade C is prevalent in India, Pakistan, Bangladesh, Myanmar, Thailand, and Malaysia and is related to *R. annulatus* [11,12,13].

Similarly, analysis of the mitochondrial 16S rDNA gene bisects *R. microplus* into two clades: clade A (isolates from India and China), which is closely related to *R. annulatus*, and clade B (isolates from Africa, America, and Southeast Asia) which is closely related to *R. australis* [14,15]. Furthermore, the proposition that *R. microplus* populations from India and China represent cryptic species [16], along with the notable diversity of haplotypes within *R. microplus s.l.* [17], underscores the need for more robust phylogenetic analyses. Therefore, this study aims to comprehensively characterize tick species infesting bovines using both newly collected data and previously published genetic sequences from the Northeastern Hill (NEH) region. Given that northern Indian isolates appear cryptic and southern Indian isolates belong to clade C, this research focuses on assessing genetic diversity using mitochondrial (COX1 and 16S rDNA) and nuclear (ITS-2 and 18S rDNA) markers. Additionally, the study seeks to identify and describe endosymbionts present in tick vectors. These endosymbionts may play important roles in the biology and ecology of the host species. Understanding both the genetic diversity and endosymbiont composition of ticks contributes to a more comprehensive understanding of the tick population in this region.

## 2. Materials and Methods

### 2.1. Sampling

**Figure 1:**
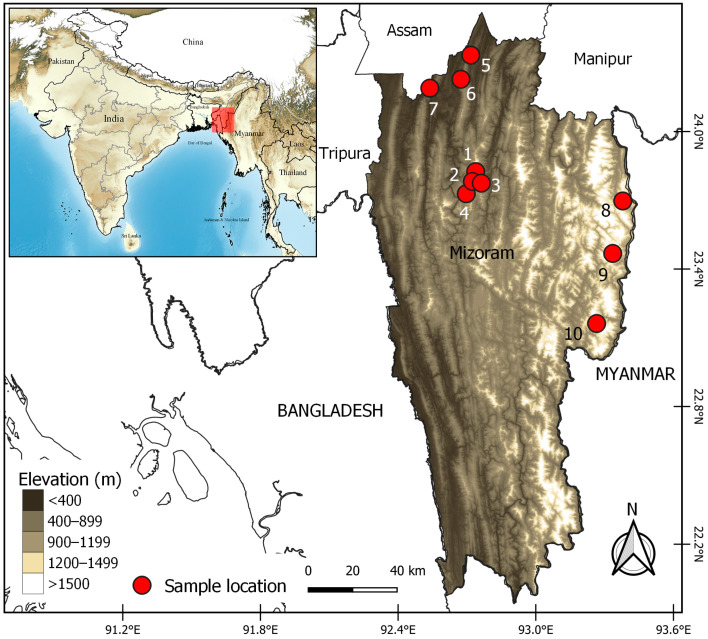
Digital elevation map showing the sample locations in Mizoram, India: 1. Sihphir, 2. Durtlang, 3. Muthi, 4. Govt. Complex, 5. Bilkhawthlir, 6. Kolasib, 7. Bairabi, 8. Hnahlan, 9. Champhai, 10. Samthang.

A total of 949 ticks were collected from Mizoram, India, which experiences a humid mild subtropical climate to humid temperate sub-alpine zones (Figure 1). The average temperature ranges from 20 °C to 29 °C in summer and 11 °C to 21 °C in winter with 2500 mm of rainfall. Tick samples were randomly collected from Zebu cattle (*Bos indicus*), Taurine cattle (*Bos taurus*), and Mithun (*Bos frontalis*) irrespective of age, sex, or breed. Ticks were carefully collected with thumb forceps from the body surfaces of animal and kept immediately in 15 mL centrifuge tube containing 70% alcohol. The collected tick samples were carried to the Department of Veterinary Parasitology, College of Veterinary Sciences and Animal Husbandry (CAU-I), Selesih, Mizoram, for examination and processing. These ticks were sorted a priori based on morphological features [18]. Before collection of tick samples from the animal, prior approval for a non-invasive mode of sample collection was obtained from the Institutional Animal Ethics Committee (IAEC) of the college.

### 2.2. DNA Isolation and PCR Amplification

DNA isolation from ticks was performed using the Genomic DNA Extraction kit (Thermo Fisher Scientific, Waltham, MA, USA) following the manufacturer’s protocol. To minimize the contamination of host DNA during its extraction, DNA was extracted from an un-engorged tick sample. Four genetic markers were selected for this study such as mitochondrial COX1 and 16S rDNA, nuclear ITS-2 (spanning between the 5.8S and 28S rDNA), and 18S rDNA with the oligonucleotide primers listed in Table 1. The PCR amplification conditions of each gene and the amplicon sizes are also mentioned in Table 1.

The PCR products were visualized on 1% (*w*/*v*) agarose gel with the addition of ethidium bromide. The PCR amplicons were subsequently purified using GeneJET Gel Extraction Kit (Thermo Fisher Scientific, Waltham, MA, USA). Following purification, the PCR products were ligated into the pTZ57R/T plasmid vector Thermo Fisher Scientific, Waltham, MA, USA and transformed into DH5α Escherichia coli cells in LB agar containing ampicillin. Positive clones, upon confirmation by colony PCR, and the stab cultures of positive clones in LB Agar in microcentrifuge tubes were sent for custom sequencing at the Department of Biochemistry, University of Delhi South Campus.

### 2.3. Detection of the Endosymbiont in Tick

To examine and characterize the endosymbionts harvested in ticks, 20 randomly selected tick DNA samples were amplified with the oligonucleotide primer pairs listed in Table 1. The samples were subjected to PCR for amplification with the following conditions, as mentioned in Table 2. One representative sample from *R. microplus* along with samples from *H. bispinosa* and *Amblyomma* spp. were selected for custom DNA sequencing. The 405 bp amplicons were purified from agarose gel and cloned into the pTZ57R/T cloning vector. Subsequently, the above-mentioned cloned amplicons were also subject to Sanger DNA sequencing.

### 2.4. Systematics and Molecular Analyses

The newly sequenced DNA fragments were preliminarily checked for their sequence quality and similarity using BioEdit software (version 7.7) and BLASTn (version 2.17.0). Separate datasets were compiled for each of the four markers in the ticks (COX1, 16S rDNA, ITS-2, and 18S rDNA) as well as for 16S rDNA for endosymbionts by combining our newly generated sequences with the published sequences retrieved from the GenBank database. Multiple sequence alignments for each DNA marker were performed using MUSCLE algorithm [23] with default parameters in MEGA 11 [24]. The DNA sequences generated in this study were submitted to GenBank and obtained accession numbers.

To refine the datasets, ambiguously aligned sites were removed using the heuristic method in trimAL software (version 1.4) [25]. Uncorrected *p*-distances were estimated in MEGA 11 [19]. The *p*-distance matrices from each dataset were standardized and utilized for Principal Coordinate Analysis (PCoA) [26]. Optimal nucleotide substitution models were determined using PartitionFinder v2.1 [27] through the Bayesian Information Criterion (BIC). Bayesian inference (BI) phylogenies were reconstructed separately for each gene using the selected nucleotide substitution models in MrBayes v3.2.5 [28]. The MCMC was run with four chains (one cold and three hot chains) for 20 million generations and sampled every 5000 generations. The first 25% of trees were discarded as burn-in and the Bayesian posterior probability (PP) values represented the nodal support for the BI tree. The maximum likelihood (ML) phylogenetic tree was also reconstructed in IQ-TREE [29] incorporating FreeRate heterogeneity [30]. The ML tree was run at 10,000 Ultrafast Bootstrap (UFB) replicates [31] using partitions determined by PartitionFinder v2.1 [27] and models selected based on BIC values by ModelFinder [32] integrated into the IQ-TREE [29]. The best nucleotide substitution models for the BI phylogenetic analyses selected by PartitionFinder v2.1 [23] were K81UF+G for 16S, GTR+G for ITS-2, K80+G for 18S, and K80+I for 16S of CLEs, while the best nucleotide substitution models selected for the ML phylogenetic analysis by ModelFinder [32] were K3Pu+F+G4 for 16S, TIM3+F+G4 for ITS-2, and K2P+G4 for 18S of ticks, and K2P+I for 16S of CLEs in the dataset. The values of estimated sample size (ESS) for the BI phylogenetic analyses in the four datasets were more than 200. The phylogenetic trees were further illustrated in iTOL software v5 [33]. The three aligned datasets of ticks were utilized for haplotype diversity assessment for determining the status of the study tick population in DnaSP v.6 [34]. The haplotype networks were plotted in PopArt v.1.7 [35] using the Median-Joining method [36].

## 3. Results

### 3.1. Systematics and Molecular Phylogeny of R. microplus

In this study, ticks infesting cattle from Mizoram were sampled, which comprised *R. microplus* and *H. bispinosa*; *Amblyomma* spp. were also collected from a species of semi-domesticated bovine, mithun. The generated DNA sequences of this study, such as COX1 (643 bp), 16S rDNA (455 bp), ITS-2 (1500 bp for *R. microplus* and 1700 bp for *H. bispinosa*), and 18S rDNA (780 bp), were submitted to GenBank (Supplementary document sheet S1).

**Figure 2:**
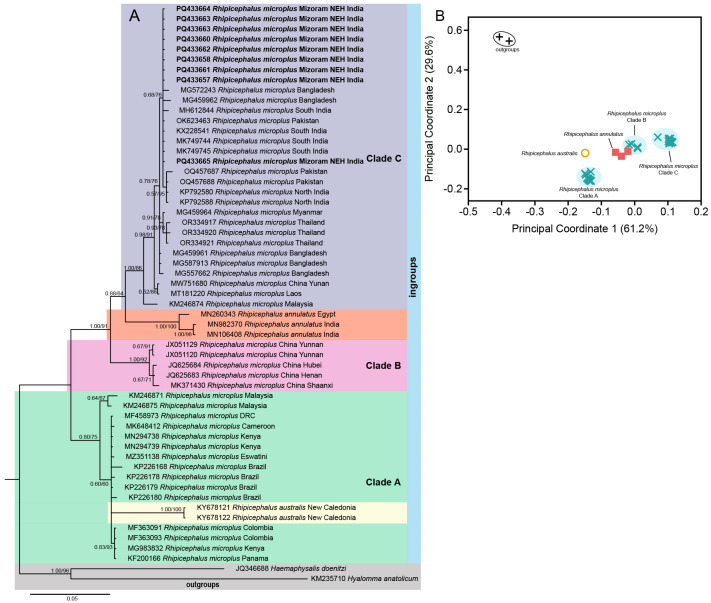
(**A**) Bayesian inference (BI) phylogenetic tree of the mitochondrial COX1 marker in assigning *R. microplus* as the ingroup while keeping *H. doenitzi* and *Hyalomma anatolicum* as outgroups. The posterior probability (PP) support values from the BI tree are given at each branch, and the ultrafast bootstrap (UFB) support for the corresponding branch from the maximum likelihood (ML) inference tree is also given beside the PP values as PP/UFB. (**B**) Ordination of standardized *p*-distance (COX1) among the ingroup and outgroup taxa along the principal coordinate (PCo) axes where a total of 61.2% and 29.6% of the variance are captured by PCo1 and PCo2, respectively.

**Figure 3:**
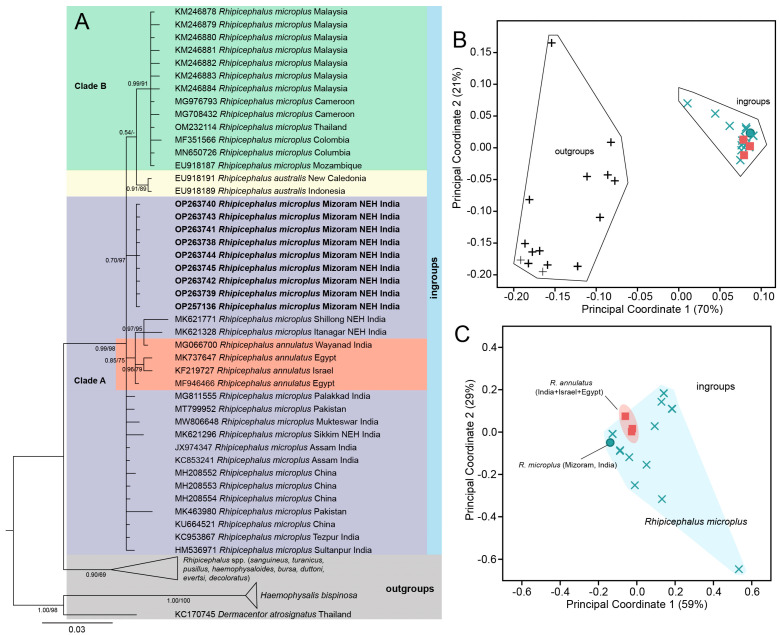
(**A**) Bayesian inference (BI) phylogenetic tree of the mitochondrial 16S rDNA marker in ixodid ticks with assigning *R. microplus* and *R. annulatus* as ingroups while keeping *H. bispinosa*, *Dermacentor* and other *Rhipicephalus* species as outgroups. The posterior probability (PP) support values from the BI tree are given at each branch, and the ultrafast bootstrap (UFB) support for the corresponding branch from the maximum likelihood (ML) inference tree is also given beside the PP values as PP/UFB. The unsupported branching from the ML tree is denoted as dash (PP/–). (**B**) Ordination of standardized *p*-distance (16S rDNA) among the ingroup and outgroup taxa along the first and second principal coordinate (PCo) axes, where a total of 70% and 21% of the variance are captured by PCo1 and PCo2, respectively. (**C**) Ordination of standardized *p*-distance among the ingroup taxa along the first and second principal coordinate (PCo) axes, where a total of 59% and 29% of the variance are captured by PCo1 and PCo2, respectively.

**Figure 4:**
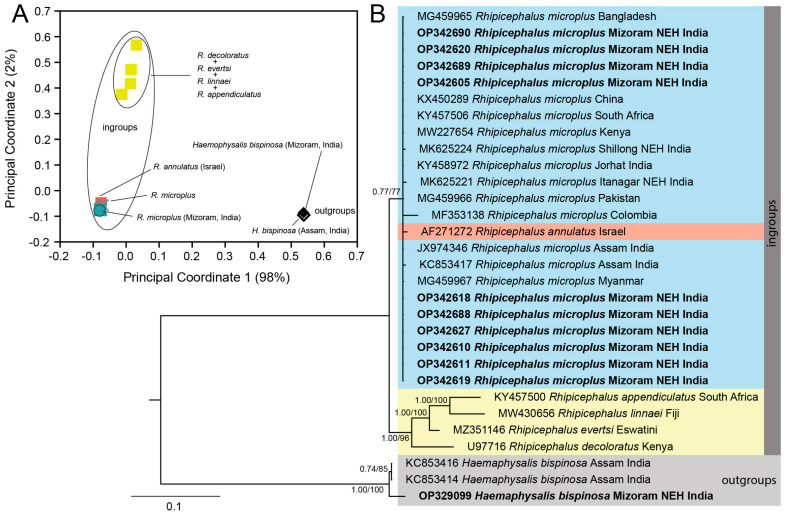
(**A**) Ordination of standardized *p*-distance (ITS-2) among the ingroup and outgroup taxa along the first and second principal coordinate (PCo) axes where a total of 98% and 2% of the variance are captured by PCo1 and PCo2, respectively. (**B**) Bayesian inference (BI) phylogenetic tree of the nuclear ITS-2 marker in ixodid ticks with *Rhipicephalus* species assigned as ingroups while keeping *Haemophysalis bispinosa* as an outgroup taxon. The posterior probability (PP) support values from the BI tree are given at each branch, and the ultrafast bootstrap (UFB) support for the corresponding branch from the maximum likelihood inference tree is also given beside the PP values as PP/UFB.

**Figure 5:**
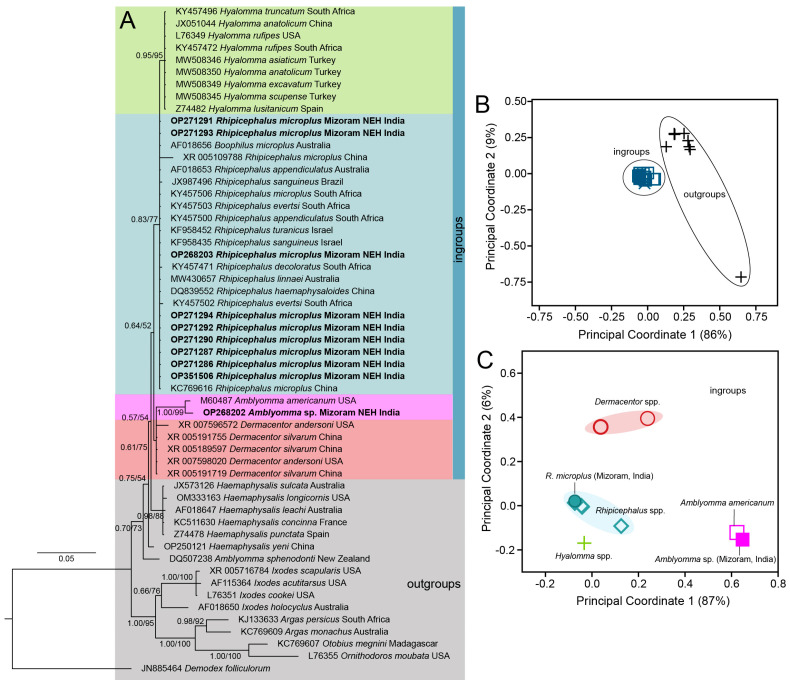
(**A**) Bayesian inference (BI) phylogenetic tree of the nuclear 18S rDNA marker in ixodid ticks with the genera *Rhipicephalus*, *Hyalomma*, *Amblyomma*, and *Dermacentor* assigned as ingroups while keeping *A. sphenodonti* and the *genera Haemophysalis*, *Ixodes*, *Argas*, *Otobius*, *Ornithodorus*, and *Demodex* as outgroups. The posterior probability (PP) support values from the BI tree are given at each branch, and the ultrafast bootstrap (UFB) support for the corresponding branch from the maximum likelihood inference tree is also given beside the PP values as PP/UFB. (**B**) Ordination of standardized *p*-distance (18S rDNA) among the ingroup and outgroup taxa along the first and second principal coordinate (PCo) axes, where a total of 86% and 9% of the variance are captured by PCo1 and PCo2, respectively. (**C**) Ordination of standardized *p*-distance among the ingroup taxa along the first and second principal coordinate (PCo) axes, where a total of 87% and 6% of the variance are captured by PCo1 and PCo2, respectively.

After trimming low-quality sequences from both ends, the aligned datasets of COX1, 16S rDNA, ITS-2, and 18S rDNA of ticks and 16S rDNA of endosymbionts (identified as Coxiella-like endosymbionts (CLEs) based on sequence similarity) consist of 849 bp, 363 bp, 729 bp, 1839 bp, and 375 bp aligned sites, respectively. Both the types of phylogenetic inferences (BI and ML) are largely concorded with each other in their topologies (Figure 2A, Figure 3A, Figure 4B and Figure 5A). For most of the cases, we employed the ordination of standardized *p*-distances (Supplementary Material sheets S2–S5) along the first two principal coordinate axes to further visualize the genetic divergence.

**Figure 6:**
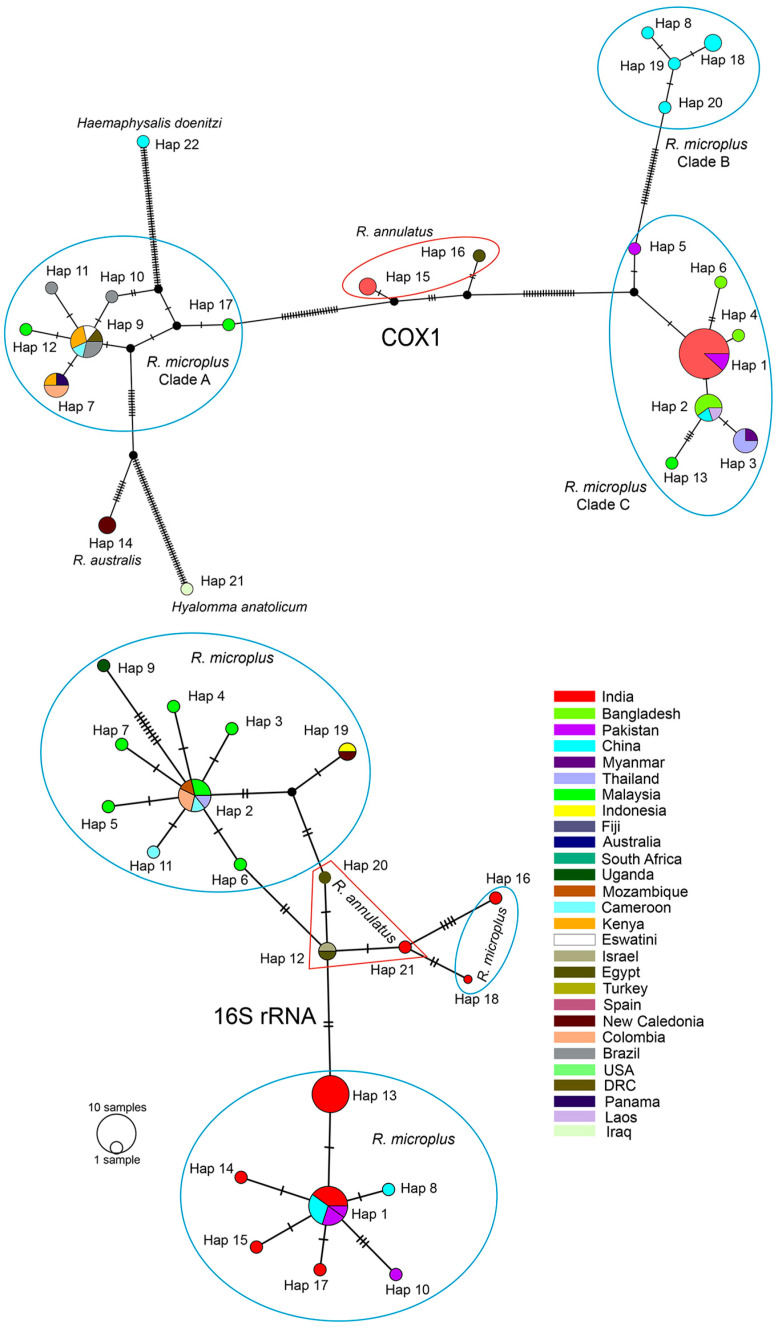
Median-joining haplotype networks based on the mitochondrial COX1 and 16S rDNA. Numbers at the branch represent mutational steps found between haplotypes, and black dots at the branch are either inferred missing or unsampled steps. The different color codes denote the different countries where the samples originate from.

In the mitochondrial COX1 datasets, *R. microplus* of NEH (Mizoram) was found to be genetically close with other isolates from north and south India, as well as neighboring countries such as Pakistan, Bangladesh, Myanmar, Thailand, Laos, China, and Malaysia. The PCoA plot revealed three clusters of *R. microplus*, corresponding to the well-supported three distinct clades in the phylogenetic tree, and the Mizoram samples clustered with respect to the other isolates (PP = 1.00; UFB = 91) in clade C (Figure 2A). Further, the clade C formed a sister clade with *R. annulatus* (PP = 0.88; UFB = 64), as opposed to the other parasites in clade A and B. In this gene fragment, we are able to identify 24 haplotypes with a haplotype diversity (hd) of 0.8774 across ingroups and 0.8531 among *R. microplus* (Table 2). The Indian isolates, including those in this investigation, are clustered into a single haplotype with Pakistani isolates (Accession No. KP792580) (Figure 3). Analyzing with the neighboring countries, three haplotypes were retrieved from Bangladesh (hap 2, 4, and 6), one haplotype from Myanmar (hap 3), and two from Pakistan (hap 3 and 5) (Figure 6).

Secondly, using PCoA, the mitochondrial 16S rDNA dataset of *R. microplus* of this study, isolates from India, certain isolates from China, and *R. annulatus* were grouped into clade A, while we assigned *R. microplus* from Malaysia, Cameroon, Thailand, Columbia, and Mozambique together with *R. australis* into clade B; and the other *Rhipicephalus* spp., *H. bispinosa* and *Dermacentor atrosignatus* as outgroups (Figure 3A). The constructed phylogenetic tree exhibited a well-supported clade that encompassed both *R. microplus* and *R. annulatus* with respect to the outgroups (PP = 0.99; UFB = 78). Notably, all of the tick samples from Mizoram (India) were clustered together, forming a sub-clade (PP = 0.70; UFB = 97). Interestingly, *R. annulatus* appeared to be cohesively clustered among *R. microplus* (Figure 3B,C). Considering this clustering pattern from PCoA, we estimated the haplotype diversity among the ingroup samples (Table 2), disclosing the existence of 21 distinct haplotypes with a haplotype diversity value (hd) of 0.8995; within *R. microplus*, 18 distinct haplotypes with an hd of 0.8804 were recovered. Intriguingly, all Mizoram samples were contained within a single haplotype (Hap 13) (Figure 6). This study retrieved seven (7) haplotypes from India, four (4) of which were from the NEH region (Hap 13, 16, 17, and 18). The haplotypes of *R. annulatus* were nested and interconnecting with *R. microplus* haplotypes from India (Hap 21), Israel (Hap 12), and Egypt (Hap 12, 20).

In the nuclear ITS-2 analysis, we assigned all the members of *Rhipicephalus* as ingroups while assigning *H. bispinosa* as an outgroup taxon considering the topology of the phylogenetic tree. The insights gained from both phylogenetic inferences and PCoA ordination showed that a cohesive cluster comprising all *R. microplus* samples, along with a single *R. annulatus* sample (Israel, Accession No. AF271272), formed a sister clade (PP = 0.77/UFB = 77) distinct from other *Rhipicephalus* species (Figure 4A,B). Correspondingly, our *H. bispinosa* sample from Bairabi, sample location no. 7 (Accession No. OP329099), clustered among the two conspecific samples from the neighboring state of India Assam, with robust branch support (PP = 1.00; UFB = 100). Among the ingroups, we determined a total of 10 distinct haplotypes with an hd of 0.5476; within *R. microplus*, 5 distinct haplotypes with an hd of 0.3377 were recovered (Table 2). Notably, all the *R. microplus* samples from Mizoram (India), along with the populations from Bangladesh (Accession No. MG459965), Pakistan (Accession No. MG459966), Myanmar (Accession No. MG459967), China (Accession No. KX450289), Kenya (Accession No. MW227654), and India (Jorhat, Accession No. KY458972; Tezpur, Accession No. JX974346; Assam, Accession No. KC853417), formed a single haplotype (Hap 1). In contrast, two Indian samples from Shillong (Hap 5, Accession No. MK625224) and Itanagar (Hap 7, Accession No. MK625221), the South African sample (Hap 2, Accession No. KY457506), and the Colombian sample (Hap 8, Accession No. MF353138) constituted separate haplotypes (Figure 4).

Regarding the nuclear 18S rDNA, based on the PCoA clusters, we assigned the genera *Hyalomma*, *Rhipicephalus*, *Amblyomma* (except *A. sphenodonti*), and *Dermacentor* as ingroups, while *Haemophysalis*, *Ixodes*, *Argas*, *Otobius*, *Ornithodorus*, *Demodex*, and *A. sphenodonti* were considered as outgroups. The resulting phylogenetic tree depicted clustering of *Amblyomma* spp. samples from Hnahlan {Figure 1 (8)} (Mizoram Accession No. OP268202) with *A. americanum* from the USA with a well-supported branch (PP = 1.00; UFB = 99) and their genetic divergence was estimated as 0.9%. Notably, *Rhipicephalus* and *Hyalomma* formed a distinct clade (PP = 0.83; UFB = 77) in contrast to *Amblyomma* and *Dermacentor*. The presence of extensive polytomy, particularly among *Rhipicephalus* species, complicated the resolution of phylogenetic relationships within this group from the available dataset (Figure 5A).

**Figure 7:**
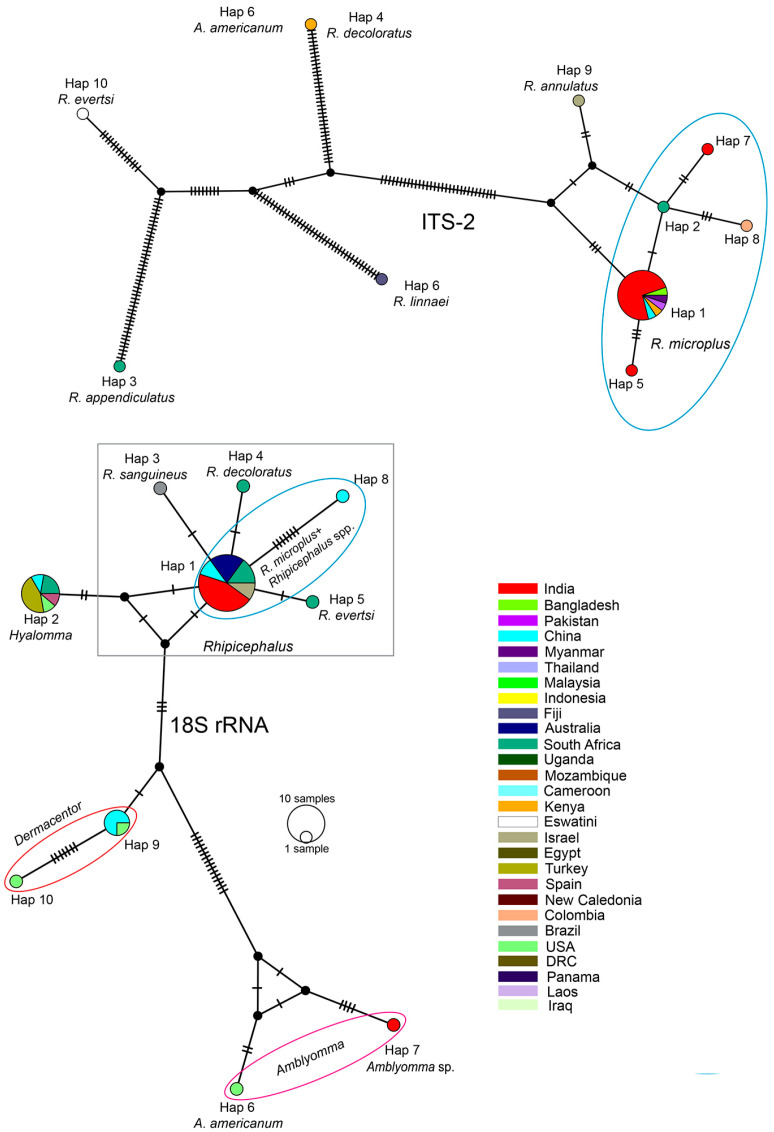
Median-joining haplotype networks based on the nuclear 18S rDNA and ITS-2 markers in ixodid ticks. Numbers at the branch represent mutational steps found between haplotypes, and black dots at the branches are either inferred missing or unsampled steps. The different color codes denote the different countries where the samples originate from.

However, when observing the ordination of the ingroup taxa on the first and second principal coordinate axes, clear clustering emerged among different ingroup taxa. For instance, the *R. microplus* population from Mizoram (India) displayed cohesive clustering with conspecific sequences. Likewise, the *Amblyomma* spp. population from Mizoram (India, Accession No. OP268202) clustered alongside *A. americanum* (Figure 5A,C). Moreover, within the ingroup taxa, a total of 10 distinct haplotypes were identified with an overall hd of 0.7026; within *R. microplus*, 2 distinct haplotypes with an hd of 0.1538 were recovered (Table 2). Except for the *R. microplus* sample from China (Hap 8, Accession No. XR_005109788), all the *R. microplus* samples from different countries and congeneric species such as *R. haemophysaloides* (China, Accession No. DQ839552), *R. appendiculatus* (Australia, Accession No. AF018653; South Africa, Accession No. KY457500), *R. sanguineus* (Israel, Accession No. KF958435), *R. turanicus* (Israel, Accession No. KF958452), R. evertsi (South Africa, Accession No. KY457503), and *R. linnaei* (Australia, Accession No. MW430657) were accommodated in a single haplotype (Hap 1). However, distinct haplotypes were formed by other *Rhipicephalus* species like *R. sanguineus* (Hap 3), *R. decoloratus* (Hap 4), and *R. evertsi* (Hap 5). Additionally, other ingroup taxa formed distant haplotypes like *Hyalomma* (Hap 2), *Dermacentor* (Hap 9, 10), *A. americanum* (Hap 6), and *Amblyomma* spp. from Mizoram, India (Hap 7) (Figure 7).

### 3.2. Detection of the Endosymbiont in Tick

Twenty randomly selected tick samples representing three species of ticks (*R. microplus*, *H. bispinosa*, and *Amblyomma* spp.) were subject to screening for the presence of endosymbionts using the 16S rDNA universal primer for prokaryotes (Table 1). Out of the 20 randomly selected tick samples, 95% (n = 19) showed amplification of the desired fragment size, at ~405 bp. From this pool, one representative amplicon from each of the three tick species was selected for subsequent custom DNA sequencing. Analyses of the DNA sequences from these representative amplicons showed 100% identity to each other. Additionally, the sequence showed a similarity of 93–99% with various *Coxiella* sp. sequences available in the public database. Notably, the DNA sequence generated in the present study showed a similarity of 95.9% with *C. burnetti*.

**Figure 8:**
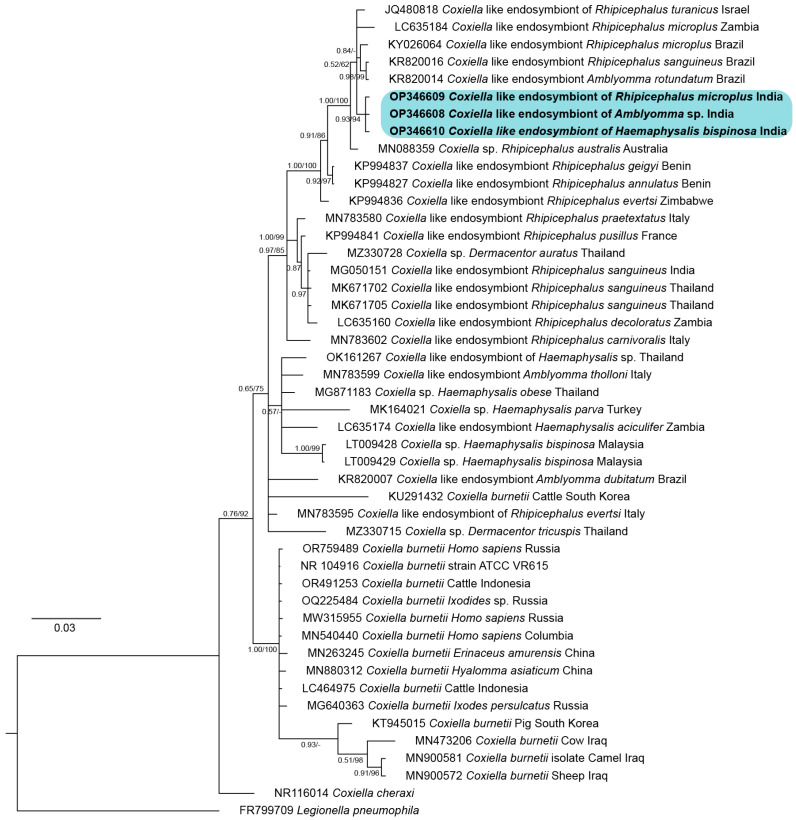
Bayesian inference (BI) phylogenetic tree of the mitochondrial 16S rDNA marker in *Coxiella* with *Legionella pneumophila* assigned as the outgroup. The posterior probability (PP) support values from the BI tree are given at each branch, and the ultrafast bootstrap (UFB) support for the corresponding branch from the maximum likelihood (ML) inference tree is also given beside the PP values as PP/UFB. The unsupported branching from the ML tree is denoted as dash (PP/–). The color shading corresponds to the sequences generated in this study.

The BI and ML trees of the *Coxiella* spp. based on the 16S rDNA fragment showed that the tick samples from Mizoram (Accession No. OP346608-10) formed a distinct cluster with a well-supported branch (PP = 0.93; UFB = 94) and were forming a subclade together with JQ480818 (*Coxiella*-like endosymbiont of *R. turanicus* from Israel), LC635184 (*Coxiella*-like endosymbiont of *R. microplus* from Zambia), KY026064 (*Coxiella*-like endosymbiont of *R. microplus* from Brazil), KR820014 (*Coxiella*-like endosymbiont of *R. rotundatum* from Brazil), and KR820016 (*Coxiella*-like endosymbiont of *R. sanguineus* from Brazil) (Figure 8). This subclade was forming a sister lineage to MN088359 (*Coxiella* sp. of *R. australis*) with a highly supported branch (PP = 1.00; UFB = 100).

## 4. Discussion

The cattle tick, *R. microplus*, is the most widely distributed tick in the world infesting bovine livestock [37,38]. This well-known parasite not only sucks blood from animals but also transmits some of the most important diseases of livestock such as babesiosis, oriental theileriosis, and anaplasmosis [39,40]. The parasite has complex taxonomic characteristics where the *R. microplus* complex includes *R. annulatus*, *R. australis*, and *R. microplus* clades A-C based on COX1 [13,16]. The COX1 marker helps in the reinstatement of *R. australis* and investigation of intraspecies variation as well as cryptic diversity which may arise from admixture of the parasite species [16]. In India, *R. microplus* clade C is found in the northern and southern states [41], and it is considered to be a cryptic species [16]. Morphological differentiation from its sister species, *R. annulatus*, can be reliably made by observing the caudal appendage in males; however, caution is advised as this feature may occasionally be absent [42]. Clade A of *R. microplus* spread to Africa and America through cattle importation [43,44]. In certain areas of southeast Asia, such as northeast Thailand and Malaysia, clade A is seen along with clade C [11,12], demonstrating the admixture of the parasite. Therefore, understanding its distribution [37,38], the diversity of various subspecies, and the pathogens and the endosymbionts it harbors will help in gaining insights into tick biology for effective tick control [45].

In the present study, tick samples were collected from Mizoram, the NEH state of India bordering Myanmar. Three types of hard ticks were identified as *R. microplus*, *H. bispinosa* and *Amblyomma* spp. based on COX1, 16S rDNA, ITS-2, and 18S rDNA; and we studied their molecular phylogeny and their haplotype diversity as well. By molecular analyses, we reaffirm that *R. microplus* in the Indian subcontinent belongs to clade C based on COX1 corroborating earlier findings from South India and neighboring countries like Bangladesh, Myanmar, and Pakistan [13,15,18,41,46,47]. Upon analysis of the partial DNA sequence of *R. microplus* based on COX1, this study revealed 23 haplotypes across the world. An integrative taxonomic study is imperative to disclose the precise species boundaries within the *R. microplus* complex. Comprehensive studies encompassing morphology, molecular analysis, and crossbreeding are of paramount importance to uncover the potential biological mechanisms that might underlie conflicting phylogenetic signals among different loci of the genome (e.g., hybridization and/or introgression, incomplete lineage sorting, etc.).

Our analysis of 16S rDNA also revealed the presence of 18 haplotypes across the world and 7 unique haplotypes specific to India, with 2 of them (hap 16 and 18) displaying closer proximity to *R. annulatus* than *R. microplus*. Moreover, the study population of *R. microplus* in this work formed a single haplotype, while the overall Indian *R. microplus* samples showed a noticeable intraspecies divergence, with a total of seven distinct matrilineal haplotypes in the 16S rDNA marker, while a single and three distinct haplotypes were seen in ITS-2 and 18S rDNA markers, respectively. These findings align with prior observations of similar polytomies identified within specific *Rhipicephalus* species groups, as highlighted by Bakkes et al. (2021) [47]. By superimposing the evidence from our phylogenetic inferences, genetic divergence, and haplotype estimation, we are convinced of the presence of cryptic diversity within *R. microplus* in India, as previously elucidated by Burger et al. (2014) [16].

Previous research has established that the evolution of the tick species experiencing pronounced diversification rates, such as the *R. sanguineus* group, is shaped by climatic conditions marked by wide annual and seasonal temperature variations [47]. Additionally, niche divergence driven by climatic conditions during dispersion episodes in off-host habitats can also contribute to limiting tick distribution and fostering speciation [48,49]. The haplotype estimation analysis conducted in the present study using 16S rDNA gene sequence aligns with this assertion by revealing a closely related matrilineal lineage between tick populations in China and India [13,16,18]. Conversely, upon analyses with the COX1 marker, the Indian (including Pakistan, Bangladesh, and Myanmar) and Malaysian isolates formed a discrete clade C [15,47], indicating a convergence among the Indian isolates based on the marker selection. The imprecise demarcation particularly notable in the Indian isolates could potentially be addressed through morpho-taxonomic studies, bio-geographic evaluation of their dispersal pattern, exploration of interbreeding tendencies with other species, expansion of sample size, and utilization of more comprehensive inferential approaches.

Systematic studies using phylogeny and haplotype networks using ITS-2 unveiled a distinct clustering of *R. microplus* and *R. annulatus* apart from other *Rhipicephalus* spp. However, this genetic marker was previously criticized for its ineffectiveness in differentiating *R. microplus* and *R. annulatus* in southern India and neighboring countries [41,47]. Burger et al. (2014) [16] recommended the exploration of an alternative nuclear marker to ITS-2. The present research found a low genetic divergence between *R. microplus* and *R. annulatus* using ITS-2 when compared to other ticks under the genus *Rhipicephalus* spp. Despite these previous studies, our research also asserts that the haplotype network of ITS-2 distinctively demarcates these sister species, demonstrating its potential for use in tick genetic diversity studies. In contrast, analysis of the 18S rDNA sequences failed to provide resolution beyond the generic level of tick in the phylogenetic inference, aligning with the observations of Mangold et al. (1998) [50]. Nevertheless, the haplotype network exhibited significant demarcation at the generic level of the ixodid ticks. This outcome also suggests the amalgamation of the *Boophilus* and *Rhipicephalus* genera (Murrell et al. 2000; Murrell & Barker 2003) [51,52].

This study also unveiled that 95% of the ticks harvested had CLEs. Instances of *C. burnetti* infection have been reported in goat-derived ixodid ticks in the north-eastern parts of India [53]. The BI tree depicted a sister lineage (putative species B) to a distinct clade comprising CLEs derived from various ticks and the pathogenic *C. burnetti*. An earlier study had shown that *Dermacentor variabilis* Kunitz-type serine protease inhibitor has a static effect against *Rickettsia montanensis* [22]. Consequently, the pathogenicity of these uncultured organisms identified as tick endosymbionts is still debatable, necessitating further comprehensive investigations.

## 5. Conclusions

This study genetically identified three tick species, *R. microplus*, *H. bispinosa*, and *Amblyomma* spp., for the first time from Mizoram, a Northeastern Hill (NEH) state in India. Based on the data we generated and recent systematic frameworks by previous researchers, we speculate that *R. microplus* s.l. dispersed across various parts of Asia through complex networks. This study also confirms that various haplotypes of the parasites circulating within India could be a cryptic species, as earlier suggested. Consequently, thorough elucidation of population genetic diversity through this study underscores the necessity of comprehensive investigations into vectors and vector-borne diseases in the north-eastern part of India. Such efforts are vital for implementing effective measures to mitigate economic losses in the livestock sector. The haplotype dispersal among *R. microplus* elucidated in this study further underscores the influence of animal transportation as a key factor driving intraspecific genetic divergence. Given that ticks are often found attached to bovine livestock, their potential to facilitate transboundary movement is significant, which could influence the transmission of ticks and tick-borne diseases (TTBDs) in the region, affecting the livestock business in the country.

## Figures and Tables

**Table 1:** List of oligonucleotide primer pairs, the target genes, PCR amplification conditions, amplicon length for the genetic characterization of various ticks, screening of haemoparasites of bovines and endosymbiont.

Target Gene	Oligonucleotide Primer	PCR Amplification Condition		Expected Amplicon Size	References
*cox*1	Forward: 5′-CTTCAGCCATTTTACCGCGA-3′ Reverse: 5′-CTCCGCCTGAAGGGTCAAA-3′	Initial denaturation	94 °C for 5 min	643	[19]
		Cyclical denaturation (35 Cycles)	94 °C for 1 min		
		Annealing temperature	56 °C for 30 s		
		Extension	72 °C for 1 min		
		Final extension	72 °C for 10 min		
		Cooling temperature	4 °C		
*16s* rDNA	Forward: 5′-AATTGCTGTAGTATTTTGAC-3′ Reverse: 5′-TCTGAACTCAGATCAAGTAG-3′	Initial denaturation	94 °C for 5 min	455	[20]
		Cyclical denaturation	94 °C for 30 s		
		Annealing temperature	49 °C for 30 s × 5 cycles		
			47 °C for 30 s × 5 cycles		
			45 °C for 30 s × 5 cycles		
			43 °C for 30 s × 33 cycles		
		Extension	72 °C for 45 s		
		Final extension	72 °C for 10 min		
		Cooling temperature	4 °C		
*ITS*-2	Forward: 5′-CGAGACTTGGTGTGAATTGCA-3′ Reverse: 5′-CCCATACACCACATTTCCCG-3′	Initial denaturation	95 °C for 10 min	1500 (*R. m*) 1700 (*H. b*)	[20]
		Cyclical denaturation (35 Cycles)	95 °C for 30 s		
		Annealing temperature	55 °C for 45 s × 33 cycles		
		Extension	72 °C for 90 s		
		Final extension	72 °C for 10 min		
		Cooling temperature	4 °C		
*18s* rDNA	Forward: 5′-CATTAAATCAGTTATGGTTCC-3′ Reverse: 5′-CGCCGCAATACGAATGC-3′	Initial denaturation	95 °C for 10 min	780	[21]
		Cyclical denaturation (35 Cycles)	95 °C for 30 s		
		Annealing temperature	52 °C for 30 s × 5 cycles		
			50 °C for 30 s × 5 cycles		
			48 °C for 30 s × 5 cycles		
			46 °C for 30 s × 33 cycles		
		Extension	72 °C for 90 s		
		Final extension	72 °C for 10 min		
		Cooling temperature	4 °C		
		Final extension	72 °C for 10 min		
		Initial denaturation	4 °C		
Endosymbiont Primer (*16s* rRNA)	Forward: 5′-GTTCGGAATTACTGGGCGTA-3′ Reverse: 5′-AATTAAACCGCATGCTCCAC-3′.	Initial denaturation	94 °C for 5 min	405	[22]
		Cyclical denaturation (35 cycles)	94 °C for 1 min		
		Annealing temperature	52 °C for 30 s		
		Extension	72 °C for 45 s		
		Final extension	72 °C for 10 min		
		Initial denaturation	94 °C for 5 min		

**Table 2:** The number of haplotypes recovered with each of the genetic markers among the ingroup and among *R. microplus* and their haplotype diversities.

Sl. No	Genetic Marker	No. of Haplotypes	Haplotype Diversity
1.	COX1 (among ingroups)	20	0.8774
2.	COX1 (among *R. microplus*)	17	0.8531
3.	16S rRNA (among ingroup)	21	0.8995
4.	16S rRNA (among *R. microplus*)	18	0.8804
5.	ITS-2 (among ingroup)	10	0.5476
6.	ITS-2 (among *R. microplus*)	5	0.3377
7.	18S rRNA (among ingroup)	10	0.7026
8.	18S rRNA (among *R. microplus*)	2	0.1538

## Data Availability

The GenBank accession numbers are provided in each of the DNA sequences generated in this study.

## Supplementary Materials

The following supporting information can be downloaded at https://www.mdpi.com/article/10.3390/genes16101216/s1.

## Abbreviations

The following abbreviations are used in this manuscript:

NEH	Northeastern Hill
COX1	Cytochrome C Oxidase sub-unit 1
PCoA	Principal Coordinate Analysis
rDNA	Ribosomal Deoxyribonucleic acid
TTBDs	Ticks and tick-borne diseases
IAEC	Institutional Animal Ethics Committee
ITS-2	Internal Transcribed Spacer-2
BIC	Bayesian Information Criterion
UFB	Ultrafast bootstrap
CLEs	Coxiella-like endosymbionts
